# *Rhopalurus junceus* scorpion venom induces antitumor effect *in vitro* and *in vivo* against a murine mammary adenocarcinoma model

**DOI:** 10.22038/ijbms.2019.33308.7956

**Published:** 2019-07

**Authors:** Alexis Díaz-García, Jenny Laura Ruiz-Fuentes, Yahima Frión-Herrera, Arianna Yglesias-Rivera, Yanelis Riquenez Garlobo, Hermis Rodríguez Sánchez, Juan C Rodríguez Aurrecochea, Ledys X López Fuentes

**Affiliations:** 1 Research Department, Laboratories of Biopharmaceuticals and Chemistries Productions (LABIOFAM), Havana, Cuba; 2 Microbiology Department, Tropical Medicine Institute “Pedro Kouri”, Havana, Cuba; 3 Department of Pharmaceutical Science, Padova University, Italy; 4 Investigation Department, Laboratory of Experimental pathology, Oncology and Radiobiology National Institute, Havana, Cuba; 5 Laboratory of Pathology, Tropical Medicine Institute “Pedro Kouri”, Havana, Cuba

**Keywords:** Antitumor, Apoptosis, Cytotoxicity, Murine mammary – adenocarcinoma, Scorpion venom

## Abstract

**Objective(s):**

In Cuba the endemic scorpion species *Rhopalurus junceus* has been used in traditional medicine for cancer treatment and related diseases. However there is no scientific evidence about its therapeutic potential for cancer treatment. The aim of the study was to determine the antitumor effect of scorpion venom against a murine mammary adenocarcinoma F3II.

**Materials and Methods:**

The cytotoxic activity was determined by MTT assay with venom concentrations ranging from 0.1–1 mg/ml. Apoptosis was determined by RT-PCR and flow cytometry. Toxic effect in healthy animals and tumor growth kinetics in F3II bearing-mice were evaluated by using scorpion venom doses (0.2; 0.8; 3.2 mg/kg) after one and ten injections respectively by the intraperitoneal route***. ***

**Results:**

Scorpion venom induced a significant cytotoxic effect (P<0.05) in F3II cells in a concentration-dependent manner. The cell death event involves the apoptotic pathway due to up-regulation of pro-apoptotic genes (p53, bax), down-regulation of antiapoptotic gene (bcl-2), and 33% of Annexin V+/PI- cells at early apoptosis and 10.21% of Annexin V+/PI+ cells at late apoptosis. Scorpion venom induced significant inhibition of tumor progression (P<0.05) in F3II bearing-mice in a dose-dependent manner. The antitumor effect was confirmed due to dose-dependent reduction of Ki-67 and CD31 proteins present in tumor tissue.

**Conclusion:**

Evidence indicates that scorpion venom can be an attractive natural product for deep investigation and developing a novel therapeutic agent for breast cancer treatment.

## Introduction

Cancer represents one of the major public health problems in Cuba and many other parts of the world. Cancer is considered to be the second cause of death around the world (1). Conventional antitumor therapy (chemotherapy, radiotherapy, and surgery), has achieved enormous advances but it is still not enough because cancer is a growing health problem (1, 2). Investigations are focused on discovering and developing new treatments based mainly on natural products, which are the most important source of new antitumor agents (3). 

Research on scorpion venoms in antitumor therapy is a current topic in this area (4). Some scorpion species like *Leiurus quinquestriatus* and *Buthus martensii karsh* have demonstrated a potential for cancer treatment which contain some toxins active on tumor cells (5, 6). In fact, the case of *B. martensii karsh* represents an interesting example because its venom has been used as traditional and folk therapy in cancer treatment and other pathophysiological conditions some centuries ago (7). 

In Cuba, the scorpion *Rhopalurus junceus* is an endemic species belonging to *the Buthidae* family (8). *R. junceus* scorpion venom has been used, in traditional medicine, for treatment of illnesses including cancer. The scorpion is widespread and its venom is not considered dangerous to human health. Additionally, it has been reported that this scorpion venom has low toxicity in mice compared to other species (9). In fact, previous reports demonstrated that *R. junceus* scorpion venom induces a selective and cytotoxic effect against a panel of epithelial human cancer cells without affecting normal cell viability (10). Despite this first report, until now, there is no scientific evidence about the antitumor effect in *in vivo* experimental models of cancer. Due to this, we studied the effect of the scorpion venom on cell viability, the mechanism of cell death and tumor progression in a murine mammary adenocarcinoma. 

## Materials and Methods

***Reagents***

Dulbecco’s modified Eagle’s medium was purchased from GIBCO/BRL (Gaithersburg, MD). Fetal bovine serum (FBS) was purchased from Hyclone. Trizol reagent was obtained from Invitrogen (Invitrogen, USA). dNTPs, GoTaq DNA polymerase, and M-MLV reverse transcriptase system were purchased from Promega (Promega Inc, USA). The 3-[4,5-dimethylth-iazol-2-yl]-2,5-diphenyl tetrazolium bromide (MTT) reagent was purchased from Sigma. All of other chemicals and reagents were obtained from Sigma (St Louis, MO).

***Venom source***

*R. junceus* scorpions were maintained in individual plastic cages in the laboratories belonging to The Entrepreneurial Group of Biopharmaceuticals and Chemistry Productions (LABIOFAM). Scorpions kept alive in the laboratory were milked by electrical stimulation for venom extraction. The venom was dissolved in double distilled water and centrifuged at 15000 rpm for 15 min. The supernatant was filtered by using 0.2 µm syringe filters and stored at -20 °C until used. The protein concentration was calculated using the modified Lowry´s method (11).

***Animals***

BALB/c male mice (18–20 g) with ages between 8–12 weeks were used. The animals were obtained from the National Center for Laboratory Animal Breeding (CENPALAB, Havana, Cuba). They were housed in plastic cages under standard conditions; food and water were administered *ad libitum*. The experimental procedure using animals was approved by the Institutional Committee for Care and Use of Laboratory Animals (Protocol 2013/3), performed in accordance with the EU Directive 2010/63/EU for animal experiments and considering the recommendations of the Guide for the Welfare and use of Animals in Cancer Research (12).

***Cell lines, reagents, and culture conditions***

The mouse F3II mammary adenocarcinoma cells and the BALB/3T3 murine fibroblast cell line (ATCC CCL163™) were used in the experiment. The cells were grown and maintained in DMEM (Sigma, St. Louis, MO) supplemented with 10% heat-inactivated FBS purchased from Hyclone, 2 mM L-glutamine, and 80 μg/ml gentamicin (Sigma, St. Louis, MO). Cells were routinely passaged using 0.25% trypsin (Sigma, St. Louis, MO) containing 0.02% EDTA (Sigma, St. Louis, MO). 

***In vitro cell viability assay (MTT assay)***

The cytotoxicity of venom against F3II cancer cells and BALB/3T3 (was determined similarly to our previous studies (10). Briefly, cells (1× 10^4 ^cells/well) were plated in 50 μl of medium/well in 96-well culture plates and scorpion venom was added at final concentrations of 0.1, 0.5, 0.5, 0.75, and 1 mg/ml in triplicate. After 72 hr of treatment, 10 μl of sterile MTT (5 mg/ml) was added. The supernatant was removed, 150 μl DMSO were added to each well and the absorbance was measured in a microplate reader at 560 nm. Wells with cells and without venom were used as controls and the experiment was repeated three times. Cell viability was expressed as the % viability, using the formula: %viability= A_560_ of treated cells/A_560_ of control cells x 100%. The IC_50_ value (venom concentration that causes 50% reduction of cells) from cancer cells was determined.

***Isolation of total RNA and RT-PCR analysis of p53, bax, bcl-2, and caspase 3 gene expressions in F3II cancer cells***

We carried out the procedure as we reported previously (13). Briefly, F3II cells (2 × 10^5^/well) seeded on 24-well culture plates were cultured for 24 hr. The ½IC_50_ value from scorpion venom was used and triplicate cell culture wells were exposed. Wells with vehicle (control cells) were included. Treated and control cell cultures were incubated for a further 8 hr, 24 hr, and 48 hr. Total RNA was isolated from cells using Trizol reagent according to the manufacturer’s specifications. The PCR amplification was carried out in a thermal cycler (AUXILAB, Spain). The primer sequences for RT-PCR were 5´-CCTTCCTGGGCATGGAGTCCTG-3’ and 5´-GGAGCAATGATCTTGATCTTC-3 for β-actin; 5´-GGGTT

AGTTTACAATCAGCCACATT-3´ and 5´-GGCCTTGAAGTTAG

AGAAAATTCA-3´ for p53; 5´-GGACGAACTGGACAGTAAC ATGG-3´ and 5´-GCAAAGTAGAAAAGGGCGACAAC-3´ for

bax; 5´-CAGGTCCTCTCAGAGGCAGATAC-3´ and 5´-CCTCT

CCAGGGACCTTAACG-3´ for bcl-2; and 5´-AGAACTTAGG

CATCTGTGGGC-3’ and 5´-ATCCAGGGGCATTGTAGCAC-3’ for caspase 3.

***Detection of apoptosis through flow cytometry***

The occurrence of apoptosis was identified with Annexin V-FITC/PI double labeling by flow cytometry. Murine breast cancer cells were added to a 24-well culture plate (1x10^5^ cells/well) and cultured at 37 ^°^C and 5% CO_2_ for 16 hr. After this period, scorpion venom ½IC_50_ was added and cell culture was incubated additionally for 48 hr before being harvested by trypsinization. The cells were washed twice with phosphate-buffered saline (PBS, pH 7.4) and centrifuged. The pellet was suspended in binding buffer (500 μl) and stained with Annexin V-FITC/PI (Sigma, USA) following the manufacturer’s recommendation. Cells were incubated for 30 min at 4 ^°^C in the dark and apoptosis was detected by flow cytometry. For each experiment, 10 000 events were recorded and the experiments were repeated twice. 

***In vivo studies***

*Evaluation of animal behavior and toxicity after scorpion venom treatment*

Toxicity test was carried out in BALB/c male mice of approximately 20±2 g body weight. Three groups of 5 animals each were injected with three scorpion venom doses (0.2; 0.8; 3.2 mg/kg) by the intraperitoneal route, in a single dose. The control group was treated with saline (NaCl, 0.9%). The animals were observed for general behavior during the first 24 hr, 5 days, and 10 days after venom inoculation. The signs and symptoms of intoxications were recorded. The experiments were repeated two times.

*Effect of R. junceus venom on tumor growth in the murine mammary adenocarcinoma F3II experimental model*

To evaluate the effect of scorpion venom on local tumor growth, F3II cells (2x10^5^ cells/0.2 ml DMEM) were injected in the subcutis of the right flank of BALB/c mice. When the tumor became palpable, mice were distributed in 4 cages at 10 mice/cage and 3 scorpion venom doses were administered (0.2; 0.8; 3.2 mg/kg) for 10 consecutive days intraperitoneally. The control group was treated with saline. The experiments were carried out for 35 days and tumor size was measured with a caliper in two dimensions (width and length) twice a week, and volume was calculated by the formula: tumor volume= [length (mm) X width^2^ (mm)] X 0.5. 

Twenty four hours after last treatment three animals from each group were sacrificed by cervical dislocation. Tumors were removed and fixed in 10% formalin and paraffin-embedded sections were stained with Hematoxylin/Eosin (H&E). Histopathological analysis of tumors was performed with tissue sections that were examined and photographed with a photomicroscope using a bright field condenser. The experiments were repeated three times.

***Western blot analysis***

After the last treatment, two mice from each group were used for tumor extraction. Tumors were obtained, dissected in small pieces and macerated in a lysis buffer (40 mM Tris-HCl pH 7.4, 10 mM EDTA, 120 mM NaCl, 1 mM DTT, 0,1% NP-40, 1% proteases inhibitors cocktail (Sigma, EUA)). The solution was centrifuged at 10000 rpm for 10 min at 4 *°C. *The supernatant was obtained and protein concentration was calculated. One hundred micrograms of total protein were separated by 10% SDS-polyacrylamide gel electrophoresis and transferred onto Hybond ECL nitrocellulose membranes (Amersham, Piscataway, NJ). After blocking with 5% skim milk for 18 hr, the membranes were washed and incubated with anti-CD31 (1:500) (DAKO, USA), anti-Ki-67 (1:500) (DAKO, USA), and anti-β-actin (1:1000) (Cell signalling, USA) for 1 hr at 37 °C. The membranes were washed and then incubated with goat anti-rabbit peroxidase-conjugated secondary antibody (1:1000) for 1 hr and bands were visualized by using the ECL Western-blotting analysis system (Amersham Piscataway, NJ).

***Statistical analysis***

The IC_50_ values were determined by tendency lines from linear regression curves. Band intensity of each gene from scorpion venom-treated and non-treated cells was compared using the Mann-Whitney U test. Differences in tumor growth under the different treatments were analyzed by using the Kruskal-Wallis non-parametric test and Dunn’s multiple comparison test. For all analysis *P*<0.05 was considered significant. For statistical analysis, we used the GraphPad Prism version 5.00 for Windows, (GraphPad Software, San Diego California, USA).

## Results

***Cytotoxic activity of scorpion venom (MTT assay)***

**Figure 1:**
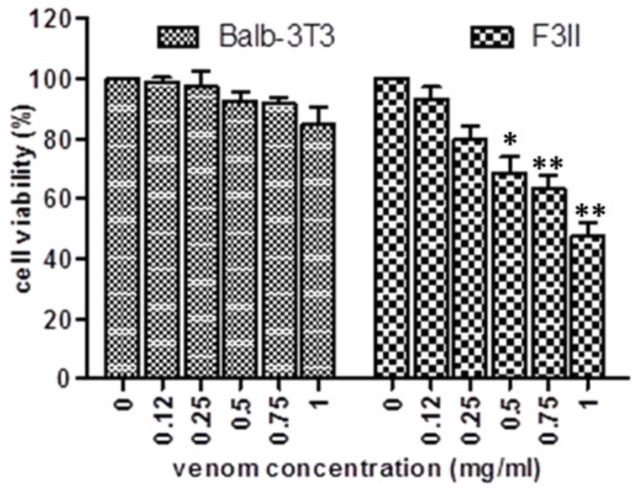
Effect of scorpion venom on cell viability of murine mammary adenocarcinoma F3II and murine fibroblast BALB/3T3. The cells were incubated for 72 hr with scorpion venom at concentrations between 0.1–1 mg/ml. The graph shows the percentage of cell viability measured by MTT and represents the mean±SE of five replicates from three independent experiments. Significant differences *P<0.05, **P<0.01 with respect to the cells without scorpion venom treatment

The exposure to scorpion venom of normal fibroblast BALB/3T3 and mammary adenocarcinoma F3II cells exerts a differential effect. After 72 hr of venom treatment in BALB/3T3 cells, there were only negligible effects on cell viability compared to control cells. Meanwhile, scorpion venom induced a significant cytotoxic effect against the F3II cancer cell line during 72 hr (*P*<0.05). The significant cell-growth inhibition was in a concentration-dependent manner (Figure 1); the IC_50_ value for F3II was 0.95±0.17 mg/ml.

***Detection of apoptosis-related genes in scorpion venom-treated cancer cells***

**Figure 2:**
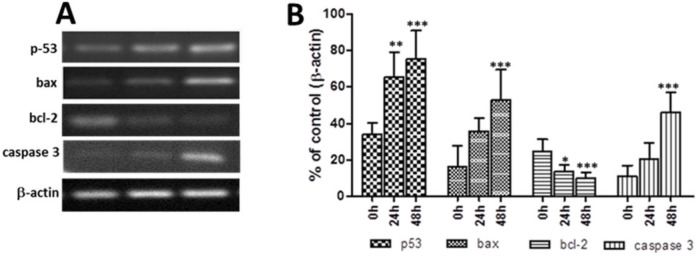
Effect of treatment with scorpion venom on the expression of apoptosis-related genes in F3II cancer cells. (A) Illustrative photograph showing the levels of the amplified gene at 0 hr, 24 hr, and 48 hr after scorpion venom treatment. β-actin was used as an internal control. (B) Graph illustrates the relative intensities of bands, representative of the expression levels of the analyzed gene. The values represent the mean ± SD of three independent experiments. Significant differences *P<0.05, **P<0.01, and ***P<0.001 with respect to the initial time (0 hr)

The effect of the scorpion venom on apoptosis-related genes p53, bax bcl-2, and caspase 3 was evaluated after 0 hr, 24 hr, and 48 hr of treatment. Figure 2 shows the amplified fragment from each gene analyzed (Figure 2A) and the kinetics of the expression levels of genes after scorpion venom treatment (Figure 2B).

The p53 gene showed a time-dependent increase that was significant after 24 hr (*P*<0.05) and 48 hr (*P*<0.001) (Figure 2B). Similarly, bax and caspase 3 genes showed a time-dependent increase which was significant at 48 hr (*P*<0.001) (Figure 2B). By contrast bcl-2 gene analysis revealed a significant decrease at 24 hr (*P*<0.05) and 48 hr (*P*<0.001) (Figure 2B). 

***R. junceus scorpion venom induces apoptosis in cancer cells***

**Figure 3:**
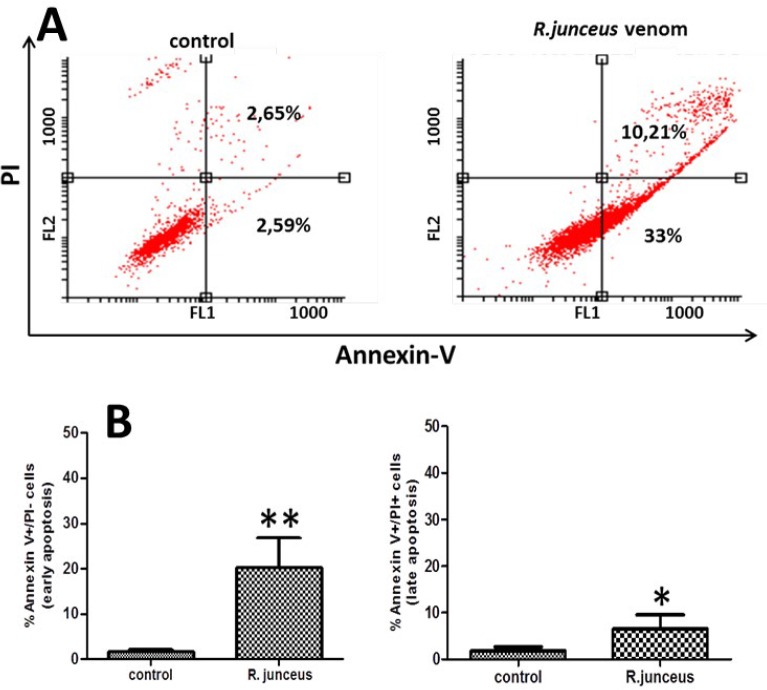
Apoptosis induction of *Rhopalurus junceus* scorpion venom on F3II cancer cells. A) Graphs represent double staining for Annexin V-propidium iodide (PI) uptake in control and scorpion venom-treated cells after 48 hr of incubation. The percentage of PI-negative and annexin V-positive cells that mean early apoptosis, and PI positive and annexin V-positive cells that mean late-stage apoptosis, are represented in each quadrant. B) Graph represents the percentages of cancer cells in early and late apoptosis stages expressed as the mean±SD of triplicate measurements. Significant differences *P<0.05, **P<0.01

The effect of *R. junceus *scorpion venom on cancer cell death was investigated by flow cytometry. The F3II cells were double labeled with Annexin V-FITC/PI to determine the cell death status of cancer cells after scorpion venom treatment. After 48 hr of scorpion venom treatment, the number of apoptotic cells at early and late apoptosis was 33% and 10.21%, respectively, with an overall behavior (early and late apoptosis) of 43.21% of cancer cells carrying out apoptotic cell death (Figure 3A). Both characteristic stages of apoptotic cell death in scorpion venom-treated cells showed significant differences with respect to untreated control cells (Figure 3B).

***R. junceus scorpion venom doses not affect the normal behavior of BALB/c mice***

Three scorpion venom doses were injected in BALB/c mice to identify the potential toxic effect. None of the scorpion venom doses caused a lethal effect in mice. Only slight discomfort was observed in the group administered with 3.2 mg/kg that disappeared two hours after venom injection.

***Antitumor effect of scorpion venom against F3II cancer cells***

**Figure 4:**
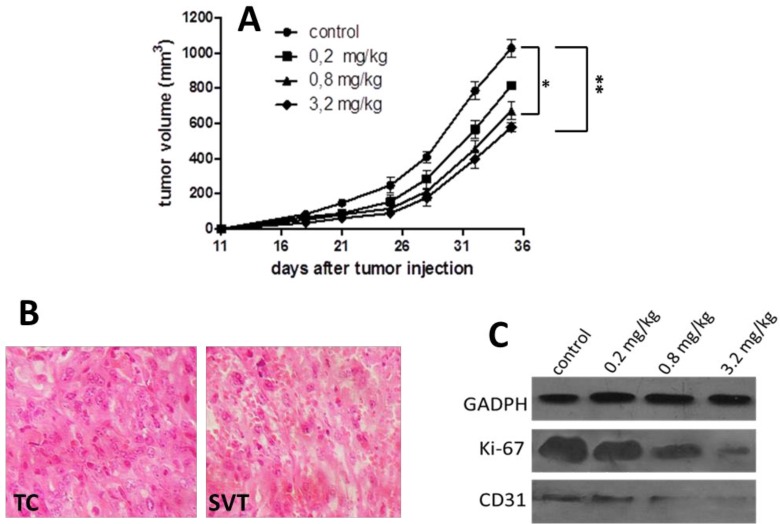
Anticancer effect of *Rhopalurus junceus* scorpion venom on F3II cancer cell bearing-mice. A) Effect of different scorpion venom doses on F3II tumor growth. Each point represents means±SD of each experimental group. Significant differences with respect to F3II-bearing mice control group, *P<0.05, **P<0.01. B) Representative micrographs from H&E staining of tumors from untreated controls (TC) displaying marked cellular and nuclear pleomorphism and cancer nest structure and scorpion venom treated group (SVT, 3.2 mg/kg) showing zones with vast necrotic areas. C) Representative Western blot showing the expression of Ki-67 and CD31 tumor markers from one tumor belonging to each experimental group in one experiment

To evaluate the effect of scorpion venom against F3II, different doses of scorpion venom were administered by the intraperitoneal route for 10 days and tumor growth was analyzed. For all treated groups there was a dose-response relationship during the 35 days considering tumor growth delay (Figure 4A). 

The 0.2 mg/kg dose only exerts a non-significant growth delay with respect to the untreated control group. Meanwhile, tumor-bearing mice treated with scorpion venom at doses of 0.8 and 3.2 mg/kg exhibited a significant tumor growth delay (*P*<0.05) compared to the untreated control group (Figure 4A).The damage in the tumor after scorpion venom treatment was evidenced by histological analysis of tumor tissue obtained 24 hr after treatment conclusion. The scorpion venom induced extended necrosis in tumor with respect to tissue from the untreated control group, which adds evidence of the antitumor properties of scorpion venom (Figure 4B). Additionally, dose-dependent reduction of Ki-67 and CD31 proteins was detected by Western blot in tumor tissue from scorpion venom-treated groups with respect to the untreated group. All six replicates analyzed in the same treatment group showed a similar behavior for each tumor marker, which confirmed the antitumor effect of *R. junceus* scorpion venom (Figure 4C). 

## Discussion

Improvement of cancer treatment involves the search for novel natural sources with anticancer potential. Scorpion venoms have shown to be suitable natural sources due to their potentialities as anticancer agents (4, 14). In this work, we demonstrated that *R. junceus* scorpion venom induces significant cytotoxicity *in vitro* against the murine mammary cancer cell line F3II in a concentration-effect relationship. Similar results were reported for *R. junceus* scorpion venom against human epithelial cancer cell lines, including breast cancer, (10) suggesting common targets in both human and murine tumors. Besides, our report corroborates the selective effect of this scorpion venom against cancer cells similar to other species (15-17). *B. martensii* *karsh* venom has shown significant cytotoxicity against brain tumor cells. At the same time, *Tytius discrepans* venom exhibits significant toxicity against the breast cancer cell line SKBR3 whilst not affecting normal cells (16). *Odontobuthus doriae* scorpion venom has shown significant toxicity against neuroblastoma cells (17). All this evidence suggests the potential of scorpion venoms for solid tumor therapy.

Under *in vitro* conditions, our findings showed that *R. junceus* scorpion induces cancer cell apoptosis. Apoptosis is a highly regulated process that is characterized by well-defined biochemical features and represents the preferred cell death event in anticancer treatment (18). As part of the apoptotic process, the P53 protein is activated and promotes its own up-regulation; which our experiments suggests is due to a significant time-dependent increase of the p53 gene. The p53-dependent apoptosis involves very early steps corresponding to the up-regulation of pro-apoptotic genes like bax and caspase 3 and down-regulation of antiapoptotic genes like bcl-2 as consequence of p53-downstream regulation (19). The increase of bax/bcl-2 rate, like our case, is considered as a key factor in the apoptotic mitochondrial pathway conducing to release of cytochrome c (Cyt c) from mitochondria (20). Cyt c is an integral part of the apoptosome, which involves the activation of caspase 9, which in turn activates the executioner caspase 3 (21). Caspase 3 recognizes and cleavages the protein Xkr8, promoting the negatively charged phospholipid phosphatidylserine (PS) exposure in the external face of the membrane, during the course of apoptosis (22). The translocation and externalization of PS, classify as one of the prominent features of apoptosis (20, 22). Besides, the external PS can specifically bind to fluorescently labeled Annexin V and this reaction is most commonly used for flow cytometric measurements of apoptosis (22). Due to this, in our experiments, flow cytometry was able to recognize a high percentage of cells undergoing apoptosis after scorpion venom treatment, confirming the results of the RT-PCR technique.

Under similar experimental conditions, previous reports have stated that epithelial human cancer cell lines undergo apoptosis after *R. junceus* venom treatment corroborated by fluorescence staining, RT-PCR, Western blot and mitochondrial membrane potential variation (10, 23). This behavior of scorpion venom, particularly against human and murine breast cancer, revealed this histological tissue as prominent for apoptotic cell death due to *R. junceus* venom anticancer treatment. Moreover, other scorpion venoms have shown to induce apoptotic cell death against cancer cells with mitochondria as key organelle in the apoptotic pathways (17, 24, 25).

Meanwhile, in *in vivo* experiments, our findings showed a significant tumor growth delay and dose-dependent decrease of Ki-67 in tumor-bearing animals treated with *R. junceus* scorpion venom supporting its potential antiproliferative properties. Ki-67 is a nuclear protein expressed during all phases of the cell cycle, except G0, and its expression has been reported to be correlated with tumor cell proliferation rate (26). Some studies have recognized that the proliferation-associated antigen Ki-67 is one of the best-known predictors of survival in patients with several malignant diseases, such as lung cancer, breast cancer, and prostate cancer (27). In particular, for breast cancer, many studies have investigated the expression of Ki-67 and suggested it as a prognostic and predictive marker (27, 28); its decreased expression has been associated with the reduction of tumor growth and efficacy of anticancer treatment (26, 28), which was already seen in our experiments. 

Similar to Ki-67, CD31 showed reduced expression in the tumor from scorpion-venom-treated mice confirming the anticancer effect of this scorpion venom against the murine breast cancer experimental model. The CD31 protein is a pan-endothelial marker in arterioles, capillaries, and venules in almost all cancers (29). The significance of CD31 expression as a prognostic marker has been stated for endometrial, cervical, pancreatic, lung, and breast cancers. All these studies concluded that angiogenesis-associated molecules like CD31 might be a useful tool as a prognostic marker in cancers (30, 31), highlighting the relevance of the antitumor effect of *R. junceus* venom, observed in this work. 

Scorpion venoms contain a variety of low-molecular-weight proteins or toxins, which are the main active components related to the biological activity (32, 33). The main targets of these toxins are the ion channels including Na^+^-channels, K^+^-channels, Ca^2+^-channels, and Cl^-^-channels (34). Ion channels are integral membrane proteins that allow the passive passage of certain ions into and out of the cell and physiologically are related to proliferation, cell signaling and migration, maintain the membrane potential, and others (35). The up-regulation of some ion channels is related to cancer hallmarks (35-37) and some scorpion toxins have anticancer properties by blocking the activity of over-expressed ion channels present in breast, colon, glioblastoma, lung, liver, hepatic, and prostate cancer (33, 38-40). Biochemical characterization of *R. junceus* venom described the presence of peptides that reversely affect the activity of ion channels that usually are related to cancer progression (9). Then, these ion channels can be suggested as a potential target for the anticancer effect of *R. junceus* venom. 

## Conclusion

*R. junceus* scorpion venom is able to induce apoptotic cell death against murine breast cancer cells *in vitro* and effectively inhibits the mammary tumor progression confirmed by Ki-67 and CD31 down-expression. Evidence indicates that this scorpion venom can be an attractive natural product for deep investigation and developing a novel therapeutic agent for breast cancer treatment. This study represents the first scientific evidence of the antitumor effect of *R. junceus* venom.
